# Medial collateral ligament laxity in valgus knee deformity before and after medial closing wedge high tibial osteotomy measured with instrumented laxity measurements and patient reported outcome

**DOI:** 10.1186/s40634-018-0164-2

**Published:** 2018-12-10

**Authors:** W. A. M. van Lieshout, C. D. Martijn, B. T. J. van Ginneken, R. J. van Heerwaarden

**Affiliations:** 1 Department of Orthopaedic Surgery, Maartenskliniek, Nijmegen, The Netherlands; 2 Centre for Deformity Correction and Joint Preserving Surgery, Kliniek ViaSana, Hoogveldseweg 1, Mill, 5451 AA The Netherlands

**Keywords:** Knee osteoarthritis, High tibial osteotomy, Valgus deformity, Laxity, Stability, Outcome

## Abstract

**Introduction:**

Medial closing wedge high tibial osteotomy (CWHTO) for valgus deformity correction was first described by Coventry whom performed an additional reefing of the medial collateral ligament (MCL) to prevent instability postoperative. In our clinic the additional reefing procedure has never been performed and instability has not been reported routinely by patients. Using instrumented laxity testing, pre- and postoperative valgus and varus knee laxity can be measured objectively. We hypothesize that absence of changes in laxity testing and subjective knee stability scores support that no additional reefing procedure is necessary.

**Materials and methods:**

In a prospective cohort study 11 consecutive patients indicated for medial CWHTO were subjected to pre- and postoperative stress X-rays in 30° and 70° of flexion and opening of the joint line was measured in degrees on the radiographs. Patient reported outcome scores were documented with the KOOS, Lysholm, SF36, Oxford Knee Score and a VAS instability scoring tool.

**Results:**

All patients (7 females) completed the study, mean age was 46 years. Mean preoperative Hip Knee Ankle angle 6.4° valgus was corrected to mean postoperative alignment 0.1° valgus. A significant difference was measured between mean pre- and postoperative 30° valgus laxity (2.8° vs 5.3°, *P* = 0.005), 30° varus laxity (6.7° vs 3.2°, P = 0.005) and 70° valgus laxity (2.0° vs 4.8°, *P* = 0.008). Postoperative patient-reported knee instability as measured with the Lysholm questionnaire was significantly improved compared to preoperative instability (*P* = 0.006). VAS instability improved, but didn’t reach significance (8.0 preoperative and 5.5 postoperative (*P* = 0.127). Other outcome measures showed improvement as well. No correlations between radiological findings and outcome scores were found.

**Conclusion:**

A significant increase in postoperative valgus laxity in 30° and 70° of flexion deems reconsidering addition of MCL reefingplasty to the medial CWHTO although patient reported outcome on subjective stability scores fails to report increase of instability in this study population. Instrumented laxity measurements of medial CWHTO patients treated with additional medial reefingplasty should be performed to prove the value of this procedure.

## Introduction

Osteotomy procedures for varus malalignment are frequently performed. Valgus malalignment deformity of the knee however, is much less frequent (2%-2,8%) (Bellemans et al. 2012) and a corrective procedure i.e. a varus producing osteotomy in these valgus knees is most often performed in the distal femur (Haviv et al. 2013; Puddu et al. 2010). Correction in the proximal (or high) tibia is much less frequent. However, if the valgus leg deformity is located in the tibia a tibial correction should be performed to prevent an abnormal knee joint line obliquity (van Egmond et al. 2017; Hofmann et al. 2004).

As an osteotomy pioneer, Coventry (1985, 1987) described the varus producing medial closing wedge high tibial osteotomy (CWHTO) to treat valgus malalignment of the knee (Coventry 1985; Coventry 1987). He stated that, by removing a bony wedge on the medial side of the proximal tibia, a laxity of the superficial medial collateral ligament (MCL) is introduced. He therefore suggests to perform a surgical reefing procedure at all times to tighten the MCL (Coventry 1985; Coventry 1987). Shoji and Insall also described this procedure in some patients (Shoji and Insall 1973). However, in our clinic no reefing procedures of the MCL are performed and patients have not reported routinely on knee instability after this procedure. Till date no study has evaluated the MCL-laxity before and after a varus producing medial CWHTO for valgus malalignment of the knee.

The MCL consists of a deep part and a superficial part that have different functions while together providing normal laxity during knee range of motion (Wymenga et al. 2006). Instrumented laxity measurements has proven that collateral laxity of the knee differs in flexion and extension in healthy subjects (Deep et al. 2015; Heesterbeek et al. 2008; Yoo et al. 2006). The collateral ligament laxity is variable in different persons and ligaments in women are more lax than in men especially in valgus stress. These reference values in healthy volunteers have been measured with knees positioned in varying flexion angles and with different types of instrumented laxity measurement methods.

Whereas laxity can be measured and quantified instability can be defined as the clinical manifestation of patient’s perceived increased laxity in the knee and is scored on subjective scales. Self-reported knee instability as well as greater dynamic varus-valgus stress are associated with worse self-reported knee confidence (Skou et al. 2014). This worse self-reported knee confidence has also been shown to predict functional decline in people with OA (Colbert et al. 2012). An absence of change to the MCL laxity as well as an unchanged sense of knee stability therefore is vital to a good outcome for a medial CWHTO.

The aim of this pilot study was to evaluate the effect of a medial CWHTO in varus-valgus laxity changes radiologically and subjectively and to report if these correlated to each other. We hypothesize that both MCL and LCL laxity do not change after a medial CWHTO, that self-reported knee instability also does not change and as a result, no additional surgical reefing procedure of the MCL is necessary.

## Material and methods

### Subjects

**Fig. 1:**
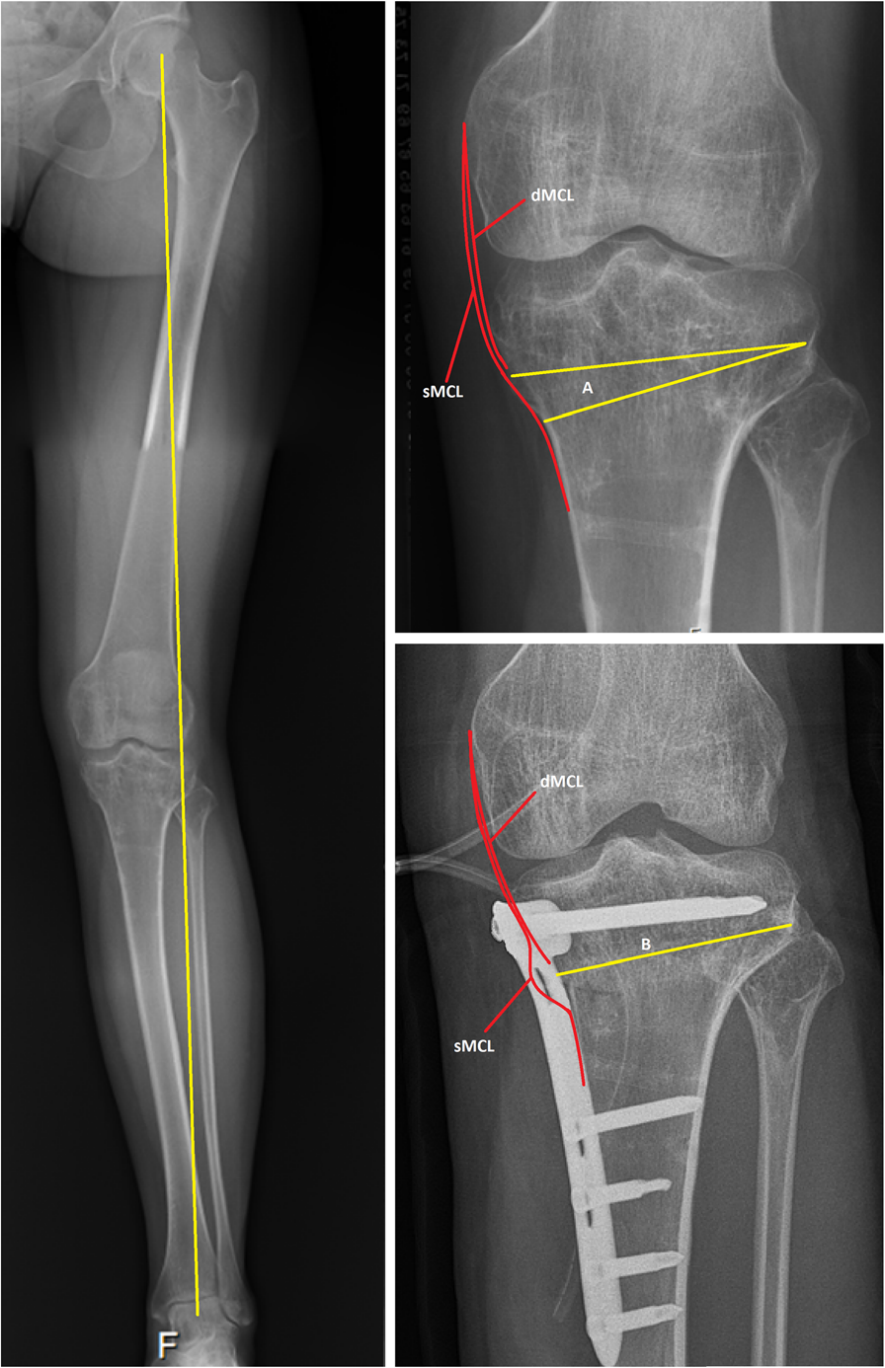
Preoperative and postoperative radiographs of the osteotomy site in relation to the medial collateral ligament. Subscript: The left figure shows a pre-operative long-leg radiographs with valgus malalignment. The right figures show a preoperative (above) and postoperative (below) radiograph of the knee. The osteotomy site (A and B) is below the insertion of the deep medial collateral ligament (dMCL). In the postoperative situation a pseudo laxity of the superficial medial collateral ligament (sMCL) is created

Between May 2015 to March 2016 eleven consecutive patients were included in this study. Inclusion criteria were symptomatic valgus malalignment located in the proximal (high) tibia, indication for a medial CWHTO, based on the severity of the complaints and the observed deformity according to Paley (Paley 2002), age 18–65 years and no history of knee ligament injuries. Exclusion criteria were previous MCL surgery, previous ipsilateral total hip replacement because of planned instrumented flexion laxity measurements, BMI higher than 30 kg/m^2^. All patients reported to the outpatient clinic and were seen and operated on by a single orthopaedic surgeon (RvH). The operative procedure consisted of surgical removal of a pre-planned bone wedge after which the created gap was closed to straighten the leg. Fixation after closure was performed with an angle stable Tomofix-plate. The location of the wedge removal is distal to the attachment of the deep part of the MCL and within the attachment of the superficial part of the MCL on the medial proximal tibia (Fig. 1). This study was approved by the Medical Research and Ethical Committee, and all patients provided written informed consent for participation in the study. Being a pilot study, no power analyses were done.

### Radiographic measurements

Pre- and postoperative long-leg radiographs were obtained for each patient. Subsequently every patient received varus and valgus stress radiographs in 30° and 70° flexion. On the day of surgery, after either spinal or total anaesthetics were applied, radiographs were made before surgery started. The postoperative radiographs were performed three to six months post-surgery in the outpatient clinic without the use of anaesthetics. At that time the subjects were instructed to relax their thigh muscles to minimize the role of the dynamic knee stabilizers when conducting the radiographs in 30° and 70° flexion in the postoperative setting.

Varus and valgus laxity of the knee in 30° flexion was assessed using the Telos device (Fa Telos, Medizinisch-Technische GmbH, Griesheim, Germany) with the subject lying in a supine position with leg muscles relaxed. The knee was positioned in an approximately 30° angle by putting a 15 cm diameter plastic pipe under the knee to ensure a reproducible angle pre- and postoperative. The Telos device was applied with 15 Nm load on the leg relative to the level of the knee joint line. While medial and lateral forces were applied, radiographs were obtained in the anteroposterior view. The direction of the X-rays was parallel to the tibia joint surface, centred on the middle of the femoral-tibial joint space.

For varus and valgus laxity of the knee in 70° flexion, a custom-made stress device was used to stress the knee and to produce reproducible measurements. An external load of 15 Nm was applied at the knee joint using 50 N on a pulley 0.30 m distal from the joint line. The knee was stressed medially and laterally. Radiographs were made with the X-ray direction parallel to the tibia joint surface in the conditions varus, valgus or no moment applied. This method has previously been described and validated by Heesterbeek et al. (Heesterbeek et al. 2008).

**Fig. 2:**
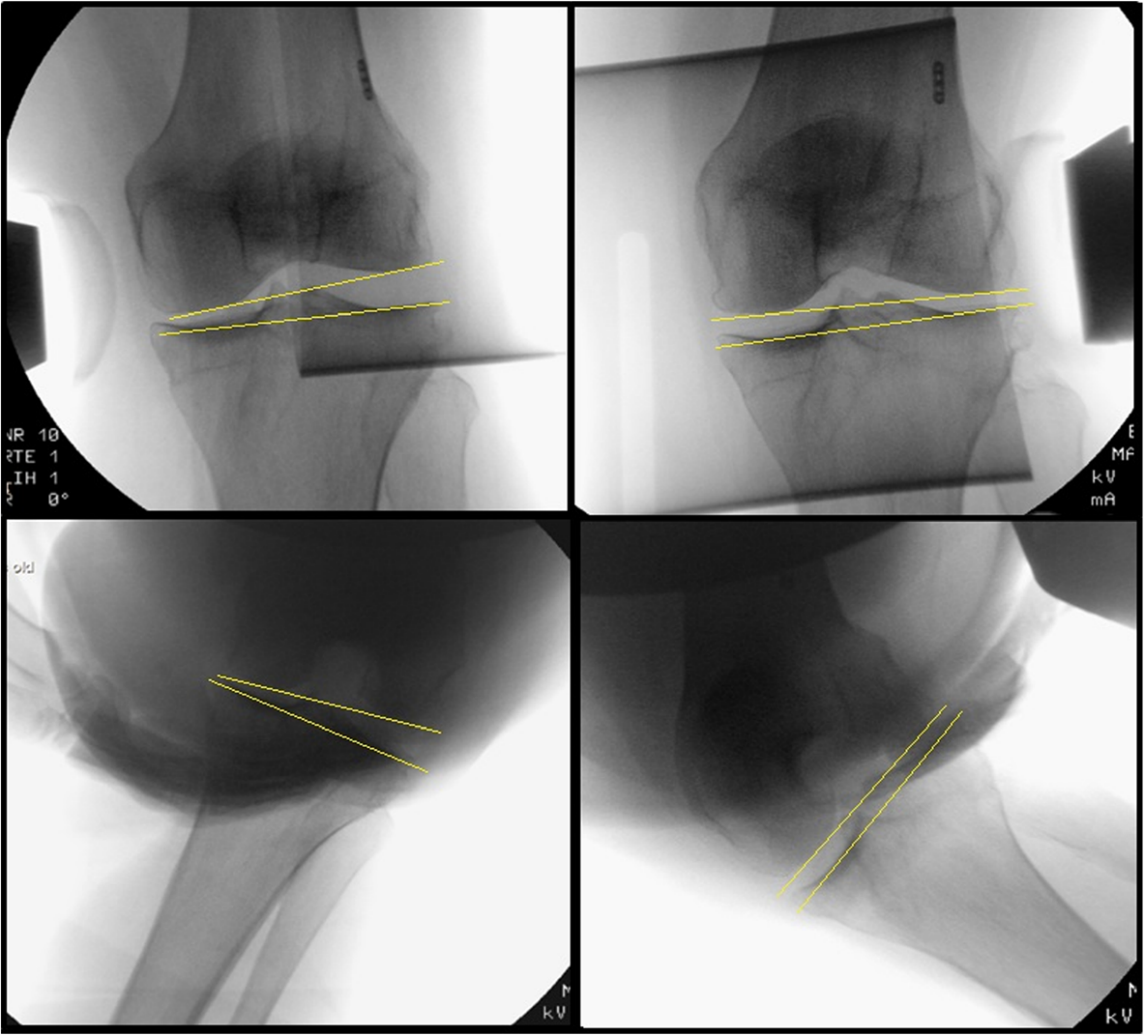
Valgus and varus laxity stress radiographs with joint space opening. Subscript: Pre-operative stress radiographs of the knee in 30° and 70° of knee flexion in an anaesthetized patient. The upper two figures show radiographs in 30° of flexion with varus (left) and valgus (right) stress. The lower two figures show radiographs in 70° of flexion in varus (left) and valgus (right) stress. The angle between a tangent line on the femur condyles and a line through the deepest tibial joint surfaces was determined and compared to the natural (unstressed) knee joint line congruence angle

The angle between a tangent line on the femur condyles and a line through the deepest tibial joint surfaces was determined on the varus, valgus and neutral radiographs using the measurement tool within the radiographic database program (Sectra workstation IDS7, version 16.1.22.1566 (2015), Sectra AB, Linköping, Sweden) (Fig. 2). Valgus laxity was defined as the difference between the medial stress radiograph and the neutral radiograph, varus laxity as the difference between the lateral stress radiograph and the neutral radiograph. The measurements were made to the nearest 0.1°. The radiographic measurements were done by two authors (WvL, CM) independently. When the independent measurements differed (> 1° apart) consensus was made. A third independent experienced orthopaedic surgeon was asked to review a sample of the measurements and reported similar measurements.

### Questionnaires

Patients were asked to fill in questionnaires preoperative and at six months postoperative. The validated Dutch Knee Injury and Osteoarthritis Outcome Score (KOOS) was used to assess subjective knee function and quality of life and consists of 5 different subscales: (1) pain, (2) symptoms, (3) function in daily living, (4) function in sport and recreation, and (5) knee-related quality of life. The score for each subscale was calculated, where 0 indicates severe knee problems and 100 indicates no knee problems (de Groot et al. 2008; Roos and Toksvig-Larsen 2003). The validated Dutch International Knee Documentation Committee (IKDC) Subjective Knee Form was used to assess symptoms and limitations in function and sports. A higher score indicates a better function (0–100 scale) (Haverkamp et al. 2006; Irrgang et al. 2001). The IKDC Current Health Assessment Form (SF36 form) was used to measure health status with respect to different dimensions: (1) physical functioning, (2) social functioning, (3) role limitations due to physical problems, (4) role limitations due to emotional problems, (5) bodily pain, (6) mental health, (7) vitality, (8) general health perception, and (10) change in perceived health during the last 12 months. All raw scores were converted to a 0–100 scale, with higher scores indicating higher levels of functioning or well-being (Haverkamp et al. 2006; Ware and Sherbourne 1992). The validated Dutch Oxford Knee Score (OKS) questionnaire was used to assess function and pain after surgery. A lower score indicates a better outcome (range 12–60) (Dawson et al. 1998; Haverkamp et al. 2005). The Lysholm knee scale was used to assess ligament injuries of the knee. A higher score indicates a better outcome (range 0–100) (Eshuis et al. 2016; Kocher et al. 2004). In addition, patients were asked to fill in a visual analogue scale (VAS) Instability, with a higher score indicating more instability of the operated knee (range 0–10).

### Statistical analysis

Differences in varus and valgus laxity of the knee in 30° and 70° flexion pre- and postoperative were assessed using nonparametric Wilcoxon signed-rank tests. Also differences in subjective scores (IKDC, Oxford, Lysholm and VAS Instability) were assessed using nonparametric Wilcoxon signed-rank tests. Results were presented as medians with range. All tests were performed with a level of significance of 0.05.

## Results

Eleven patients were included in this study, with a median age at the time of operation of 46 years (range 24–66 years). One patient was 66 at the time of operation but was accepted for this study as she was still 65 years old when put on the waiting list for this surgery. Seven patients were female and 4 were male. In one patient preoperative 30° flexion X-rays could not be obtained due to a technical error. All eleven patients completed the study. Preoperative mean hip-knee-ankle (HKA) angle was 6.4° valgus (12° valgus to 1° valgus) and this was restored postoperative to a mean HKA angle of 0.1° (5.8° valgus to 5° varus). In one patient the postoperative long leg radiograph was missing.

### Stress X-rays in 30° and 70° flexion

After separate radiological assessment by the two authors consensus had to be made for 6 patients regarding at least one value. Pre- and postoperative measurements for every single stress X-ray group are presented in Table 1. Overall, in the preoperative setting patients had higher varus laxity values compared to valgus laxity in both 30° and 70° flexion. Postoperative, this differentiated collateral laxity of knee shifted to a more distinguished valgus laxity and a decrease in varus laxity of the knee. This indicated tighter knees on the lateral side and increased laxity on the medial side of the knees.

**Table 1:** Differences in varus and valgus laxity in 30° and 70° flexion of the knee

	Preoperative*	Postoperative*	*P* value
30° Valgus Laxity	2.8° (−4.0–4.3°)	5.3° (2.5° – 7.1°)	0.005*
30° Varus Laxity	6.7° (3.7° – 9.3°)	3.2° (− 0.9° – 4.9°)	0.005*
70° Valgus Laxity	2.0° (−2.6° – 5.4°)	4.8° (0.4° – 7.6°)	0.008*
70° Varus Laxity	3.8° (− 3.2° – 9.0°)	1.3° (− 0.1° – 8.3°)	0.113

Values are presented as: median (range)

* Significant difference (*P* < 0.05)

When comparing the pre- and postoperative results for each condition, there was a statistically significant difference between the pre- and postoperative 30° valgus laxity (respectively 2.8° and 5.3° (*P* = 0.005)), 30° varus laxity (respectively 6.7° and 3.2° (P = 0.005)) and 70° valgus laxity (respectively 2.0° and 4.8° (*P* = 0.008)). The difference between the pre- and postoperative 70° varus laxity did not reach significance (3.8° versus 1,3° (*P* = 0.113)).

### Patient reported outcome scores

Patient-reported outcome results are presented in Tables 2, 3, and 4. Postoperative patient-reported knee instability as measured with the Lysholm questionnaire was significantly improved compared to preoperative instability (*P* = 0.006, Table 2). The VAS knee instability score also improved, but didn’t reach significance (8.0 preoperative and 5.5 postoperative (*P* = 0.127, Table 2)). Knee function, knee pain and knee-related quality of life as measured with the IKDC Knee evaluation form, Oxford Knee Score and the KOOS all showed significant improvements postoperative as shown in Table 3. Only the quality of life domains ‘Physical functioning’ and ‘Bodily pain’ of the IKDC Current Health Assessment Form showed significant improvements (Table 4).

**Table 2:** Differences in patient-reported knee instability as measured with the Lysholm and VAS

	Preoperative	Postoperative	P value
Lysholm	42 (26–75)	67 (27–90)	0.006*
VAS knee instability	8.0 (2.5–9.0)	5.5 (1.0–8.0)	0.127

Values are presented as: median (range)

* Significant difference (*P* < 0.05) Abbreviations: *VAS* Visual Analogue Scale

**Table 3:** Differences in knee function, pain and knee-related quality of life as measured with PROMs

	Preoperative	Postoperative	P value
IKDC Knee Evaluation	32 (13–51)	60 (24–82)	0.004*
Oxford Knee Score	35.0 (20–47)	23.5 (15–51)	0.017*
KOOS Pain	42 (17–75)	72 (22–92)	0.041*
KOOS Symptom	43 (12–86)	68 (54–96)	0.007*
KOOS ADL	53 (21–90)	81 (35–97)	0.008*
KOOS Sport & Recreation	0 (0–25)	35 (0–70)	0.020*
KOOS QoL	25 (6–50)	38 (6–63)	0.044*

Values are presented as: median (range)

* Significant difference (*P* < 0.05) Abbreviations: *PROMs* patient reported outcome measures, *IKDC* International Knee Documentation Committee; *KOOS* Knee Injury and Osteoarthritis Outcome Score, *ADL* activities of daily living, *QoL* Quality of life

**Table 4:** Differences in health-related quality of life as measured with the IKDC Current Health Assessment Form

	Preoperative	Postoperative	P value
Physical functioning	55.0 (5–85)	75.0 (40–95)	0.020*
Physical role functioning	62.5 (0–100)	87.5 (0–100)	0.357
Bodily pain	51.0 (0–74)	62.0 (41–84)	0.014*
General Health	72.0 (52–97)	82.0 (52–95)	0.420
Vitality	65.0 (50–90)	65.0 (55–90)	0.475
Social functioning	88.0 (38–100)	100.0 (50–100)	0.596
Emotional role functioning	100.0 (67–100)	100.0 (0–100)	0.157
Mental Health	88.0 (52–100)	88.0 (68–100)	0.157

Values are presented as: median (range)

* Significant difference (*P* < 0.05)

No correlations were found between radiological findings and patient reported outcome scores.

## Discussion

This study has shown a significant increase in valgus laxity in both 30° and 70° of flexion after medial closing wedge HTO, which indicates a change of laxity of the medial collateral ligament. Furthermore, a significant decrease in varus laxity in 30° of flexion and a non-significant decrease in 70° flexion, was found. These findings are not correlated to subjective knee stability and outcome scores which all improved after the operation. Therefore, our hypothesis is false.

Our preoperative valgus and varus laxity values in 70° of flexion are comparable with the normal values in healthy individuals. Our reported valgus and varus laxity in 70° flexion are 2.0° and 3.8° versus 2.5° and 3.1° as found in the study of Heesterbeek et al. who performed instrumented laxity measurements with the same measurement device and are therefore comparable (Heesterbeek et al. 2008). In a study conducted by Deep the varus and valgus laxity was measured with instrumented laxity measurements in 15° flexion in healthy individuals. This study showed that the femorotibial mechanical angle (FTMA) changed with a mean of 6.9° when 10 Nm varus torque was applied. With valgus torque the mean change in FTMA was 7.9°. The unstressed FTMA was 1.2° varus (Deep 2014). Our reported varus and valgus laxity in 30° flexion preoperative were respectively 6.7° and 2.8°. Our values are therefore lower than reported by Deep, however, we performed the tests in 30° of flexion. This might influence the results.

Our postoperative results cannot be compared to the literature since this is the first study to our knowledge to investigate the change in knee laxity after a medial CWHTO. Moreover, due to the difference in radiological measurement settings (i.e. under anaesthetics preoperative and non-anaesthetized conditions postoperative) we have possibly inflicted a non-comparable situation especially as regards the varus laxity measurements. Tsukeoka and Tsuneizumi proved that anaesthesia significantly influenced knee joint laxity on both the medial and lateral side after TKA. They reported a positive correlation between the laxity under anaesthesia and the amount of change in laxity on the lateral side (*r* = 0.57; *p* = 0.0022). They found an increase in laxity for varus stress with mean of 1° under anaesthetics in a 15 degree flexion angle of the knee. Moreover, in 23% of their patients they reported an increase of > 3° in laxity measured under anaesthesia as compared to non-anesthetized patients (Tsukeoka and Tsuneizumi 2016). The decrease of varus laxity comparing the postoperative non-anesthetized results to the preoperative anesthetized results may be contributed solely to the effect of anaesthesia and may in fact display the same unchanged lateral collateral laxity. Therefore, the different measurement setting is a plausible explanation for the unexpected decrease in varus laxity in this patient population.

However, in valgus laxity there is an increase in laxity postoperative even though the postoperative X-rays were performed without the use of anaesthetics. Therefore, the difference in measurement settings cannot explain the increased valgus laxity. A previous study by Mains et al. studied the influence of the separate ligaments with regards to stability of the knee. They have shown that the superficial MCL (sMCL) is the main stabilizer for valgus stress. After sectioning the sMCL the abduction increased with 4.8° in 20–25° of knee flexion and for 40–45° of knee flexion this increase in abduction was 4.3° (Mains et al. 1977). These findings are in correspondence with our increase in valgus laxity after the operation. Hence, we noted less increase in valgus laxity but this may again be explained due to the different setting during the radiological measurements (anaesthesia or not).

An explanation for the increase in valgus laxity can be found in the surgical technique for medial CWHTO. The MCL is composed of a superficial and a deep component originating from the medial epicondyle of the femur. The deep component inserts directly into the edge of the tibia plateau and therefore will not be affected by the osteotomy. Mains et al. showed that the deep MCL tightens at 45° flexion but is less powerful as a stabilizing factor for valgus stress compared to the superficial MCL (Mains et al. 1977). Griffith et al. showed that cutting the deep MCL resulted in increased valgus laxity in 60 degrees of knee flexion. This was not recorded for 0, 20, 30 or 90 degrees of knee flexion (Griffith et al. 2009). The superficial component is attached to the tibia approximately 6 cm beneath the joint line and has a broad insertion up to 12 cm below the joint line (LaPrade et al. 2007). The superficial MCL provides the primary resistance against valgus stress and external rotation (Griffith et al. 2009; Mains et al. 1977). When performing the medial CWHTO part of the sMCL is pushed dorsal at the location of the osteotomy when inserting a retractor subperiostially around the posteromedial tibia to protect the soft tissues while performing the osteotomy. Although care is taken not to loosen the MCL fibres during insertion of the retractor the removal of the bone wedge and the gap closure afterwards causes a relative lengthening of the MCL similar to the amount of bone resected on the medial cortex as the insertion of the sMCL remains unchanged (Fig. 1). So the superficial component fibers themselves have the same length but their underlying surface is shortened due to the removed bony wedge resulting in a pseudo laxity of the MCL and thereby introducing increased valgus laxity.

The documented significant changes in laxity in the coronal plane have to be compared with the patient reported outcome score, to identify whether they have any clinical relevance. Patients experience walking with a deformed leg, whether valgus or varus, often as an instability in walking. Patient reported outcome measures after the medial CWHTO showed significant less knee instability and knee instability-related problems 6 months after the surgery that corrected their leg to neutral as measured with the Lysholm scoring tool. The VAS instability score, solely developed to assess the patients subjective stability feeling of the knee, also improved postoperative however, did not reach significance. Due to our short follow up time (6 months) no comparisons can be made with previous studies (Coventry 1987, Shoji and Insall 1973, Chambat et al. 2000, Van Egmond et al. 2017) that reported on outcome after an medial CWHTO. Furthermore, no long-term conclusions can be drawn based on our outcome scores. However, the  findings do support our hypothesis that patients do not experience a disadvantage of the increase in laxity of the MCL. In our department as yet unpublished data among 113 comparable patients showed a 77% satisfaction after a medial CWHTO with a mean follow up of four years (van Lieshout WAM et al. n.d.).

Our study has several limitations. First, because this study is a pilot study the sample size is small. Nevertheless we reached significant changes for varus and valgus laxity for pre- and postoperative results. Second, the fact that the preoperative laxity measurements were performed after either spinal or total anaesthetics and the postoperative measurements in a normal non-anesthetized state is a confounding factor as mentioned before. However, as we thought it unethical to re-apply spinal or total anaesthesia for the sole purpose of laxity measurements, this was the only way to perform the present study in our patients. Thirdly, our patient reported outcome scores were only available at six months postoperative. Therefore no long-term conclusion regarding outcome can be drawn from them. We conducted these surveys to evaluated patient reported instability before and after medial CWHTO. Since full-weight bearing without the use of walking aids was allowed after 4 weeks we believe that a normal walking pattern and reliable patient reported stability complaints can be obtained after 6 months. A last remark has to be made why we choose to perform a medial CWHTO. Our inclusion criteria were symptomatic valgus malalignment located in the proximal (high) tibia for which an indication for a medial CWHTO is the treatment of choice as deformities localized in the tibia need to be corrected in the tibia. In distal femoral valgus corrections the osteotomies are performed proximal of collateral ligaments attachments and no changes in ligament laxity will result from corrections in the femur. However, when a distal femur correction is performed in a patient with valgus localized in the tibia an unintended obliquity of the knee joint line will be created which may cause new complaints.

In conclusion, this study shows a significant increase in postoperative valgus laxity in 30° and 70° of flexion after medial closing wedge HTO which deems reconsidering addition of a MCL reefingplasty to the medial CWHTO although patient reported outcome on subjective stability scores fails to report increase of instability in this study population. Instrumented laxity measurements of medial CWHTO patients treated with additional medial reefingplasty should be performed to prove the value of this procedure.

## Abbreviations

CWHTO: Closing wedge high tibial osteotomy
dMCL: Deep portion of the medial collateral ligament
FTMA: Femoral tibial mechanical angle
IKDC: International Knee Documentation Committee
KOOS: Knee Injury and Osteoarthritis Outcome Score
LCL: Lateral collateral ligament
MCL: Medial collateral ligament
OKS: Oxford knee score
sMCL: Superficial portion of the medial collateral ligament
VAS: Visual analogue scale
